# Accelerated mineral bio-carbonation of coarse residue kimberlite material by inoculation with photosynthetic microbial mats

**DOI:** 10.1186/s12932-023-00082-4

**Published:** 2023-06-16

**Authors:** Thomas Ray Jones, Jordan Poitras, Emma Gagen, David John Paterson, Gordon Southam

**Affiliations:** 1 School of Earth & Environmental Sciences, The University of Queensland, St. Lucia, QLD, 4072 Australia; 2 The Australian Synchrotron (ANSTO), Clayton, VIC 3168 Australia

**Keywords:** Kimberlite, Mineral carbonation, Photosynthetic biofilm

## Abstract

Microbiological weathering of coarse residue deposit (CRD) kimberlite produced by the Venetia Diamond Mine, Limpopo, South Africa enhanced mineral carbonation relative to untreated material. Cultures of photosynthetically enriched biofilm produced maximal carbonation conditions when mixed with kimberlite and incubated under near surface conditions. Interestingly, mineral carbonation also occurred in the dark, under water-saturated conditions. The examination of mineralized biofilms in ca. 150 µm-thick-sections using light microscopy, X-ray fluorescence microscopy (XFM) and backscatter electron—scanning electron microscopy-energy dispersive x-ray spectrometry demonstrated that microbiological weathering aided in producing secondary calcium/magnesium carbonates on silicate grain boundaries. Calcium/magnesium sulphate(s) precipitated under vadose conditions demonstrating that evaporites formed upon drying. In this system, mineral carbonation was only observed in regions possessing bacteria, preserved within carbonate as cemented microcolonies. 16S rDNA molecular diversity of bacteria in kimberlite and in natural biofilms growing on kimberlite were dominated by Proteobacteria that are active in nitrogen, phosphorus and sulphur cycling. Cyanobacteria based enrichment cultures provided with nitrogen & phosphorus (nutrients) to enhance growth, possessed increased diversity of bacteria, with Proteobacteria re-establishing themselves as the dominant bacterial lineage when incubated under dark, vadose conditions consistent with natural kimberlite. Overall, 16S rDNA analyses revealed that weathered kimberlite hosts a diverse microbiome consistent with soils, metal cycling and hydrocarbon degradation. Enhanced weathering and carbonate-cemented microcolonies demonstrate that microorganisms are key to mineral carbonation of kimberlite.

## Highlights

- Kimberlite-water interactions supports the growth of biofilm.
- Light-limited evaporite conditions produced gypsum, limiting mineral carbonation.
- Kimberlite weathering was enhanced by adding a cultivated photosynthetic biofilm.
- Biogeochemical activity is responsible for accelerated mineral carbonation of kimberlite.

## Introduction

The role of microorganisms within the mining and environmental industries has increased significantly in recent decades [4, 10, 23]. With an increasing expectation of companies to lower their carbon footprint, biotechnological methods that enhance extraction and processing may also provide novel approaches for mineral carbonation. Understanding the fundamental microbe-mineral interactions occurring in mined ultramafic material are essential to accelerate bioweathering and microbial carbonate precipitation within these materials, e.g., kimberlite residue remaining after diamond extraction is complete.

Microorganisms can enhance mineral weathering, impacting the environments they inhabit via the extraction of nutrients from their surroundings [24]. Physiological processes that enhance weathering include the formation of organic and inorganic acids that work to break down minerals, as well as the production of bicarbonate ions, which alters pH. Kimberlite mines provide an excellent natural laboratory to study weathering, bacteria-mineral interactions, and mineral carbonation reactions that sequester atmospheric CO_2_.

Under alkaline conditions, consistent with kimberlite, i.e., ultramafic rock, the CO_2_ equilibrium will shift geochemically towards carbonate precipitation via a bi-carbonate step (Eqs. 1, 2 and 3):

1 $${{\mathrm{CO}}_{2}}_{(g)}+{{\mathrm{H}}_{2}\mathrm{O}}_{(l)} \leftrightarrow {{\mathrm{ H}}_{2}{\mathrm{CO}}_{3}}_{(aq)}$$

2 $${{\mathrm{H}}_{2}{\mathrm{CO}}_{3}}_{(aq)}+ {{\mathrm{OH}}^{-}}_{(aq)}\leftrightarrow {{\mathrm{HCO}}_{3}^{-}}_{(aq)}+ {{\mathrm{H}}_{2}\mathrm{O}}_{(l)}$$

3 $${{\mathrm{HCO}}_{3}^{-}}_{(aq)}+ {{\mathrm{OH}}^{-}}_{(aq)} \leftrightarrow {{\mathrm{CO}}_{3}^{2-}}_{(aq)}+ {{\mathrm{H}}_{2}\mathrm{O}}_{(l)}$$

In this system, autotrophic, photosynthetic microorganisms can serve as a conduit for carbon mineralisation (Eq. 4) via the release of $\left({OH}^{-}\right)$ [31], which shifts the equilibrium towards the draw-down of CO_2_ into boundary layer water (Eqs. 1, 2 and 3). Kimberlites also supply appropriate cations during weathering, i.e., calcium (Ca) and magnesium (Mg) (Eq. 5; [3], which are preferentially precipitated as carbonates (Eq. 6; [8]:

4 $${{6\mathrm{ HCO}}_{3}^{-}}_{(\mathrm{aq})}+{{6\mathrm{ H}}_{2}\mathrm{O}}_{(\mathrm{l})}\stackrel{\upgamma }{\to } {{\mathrm{C}}_{6}\mathrm{H}}_{12}{\mathrm{O}}_{6(\mathrm{biomass})}+ {{6\mathrm{ O}}_{2}}_{(\mathrm{g})} + {{6\mathrm{ OH}}^{-}}_{(\mathrm{aq})}$$

5 $${\mathrm{Me}}_{\mathrm{x}} {{\mathrm{SiO}}_{(2+\mathrm{x})}}_{(\mathrm{s})}+2{{\mathrm{CO}}_{2}}_{(\mathrm{g})}+\mathrm{x }{{\mathrm{H}}_{2}\mathrm{O }}_{(\mathrm{l})} \to {{\mathrm{xMe}}^{(2+)}}_{(\mathrm{aq})}+2{{\mathrm{HCO}}_{3}^{-}}_{(\mathrm{aq})}+\mathrm{x }{{\mathrm{SiO}}_{(2)}}_{(\mathrm{s})}$$

6 $${\mathrm{Ca}}^{2+}+ {{\mathrm{HCO}}_{3}^{-}}_{(\mathrm{aq})}+ {{\mathrm{OH}}^{-}}_{(\mathrm{aq})}\to {\mathrm{CaCO}}_{3(\mathrm{s})}+ {{\mathrm{H}}_{2}\mathrm{O}}_{(\mathrm{l})}$$

An inevitable condition occurring during the successive disposal of mined kimberlite residue into storage piles is the deposition of fresh, wet material on top of older, often drier material, resulting in the formation of a layered surface deposit. As this environment changes, e.g., wetting/drying occurring after burial, so too will the microbiome inhabiting this environment. These physical changes will result in a shift in the dominant metabolic activity, from phototrophic to lithotrophic/heterotrophic, thus altering the metabolic products and biogeochemical activity in these ecosystems. The complexity, and therefore biodiversity of these environments is increased by the occurrence of microenvironments, where biogeochemical activity can occur on the scale of bacteria.

**Fig. 1:**
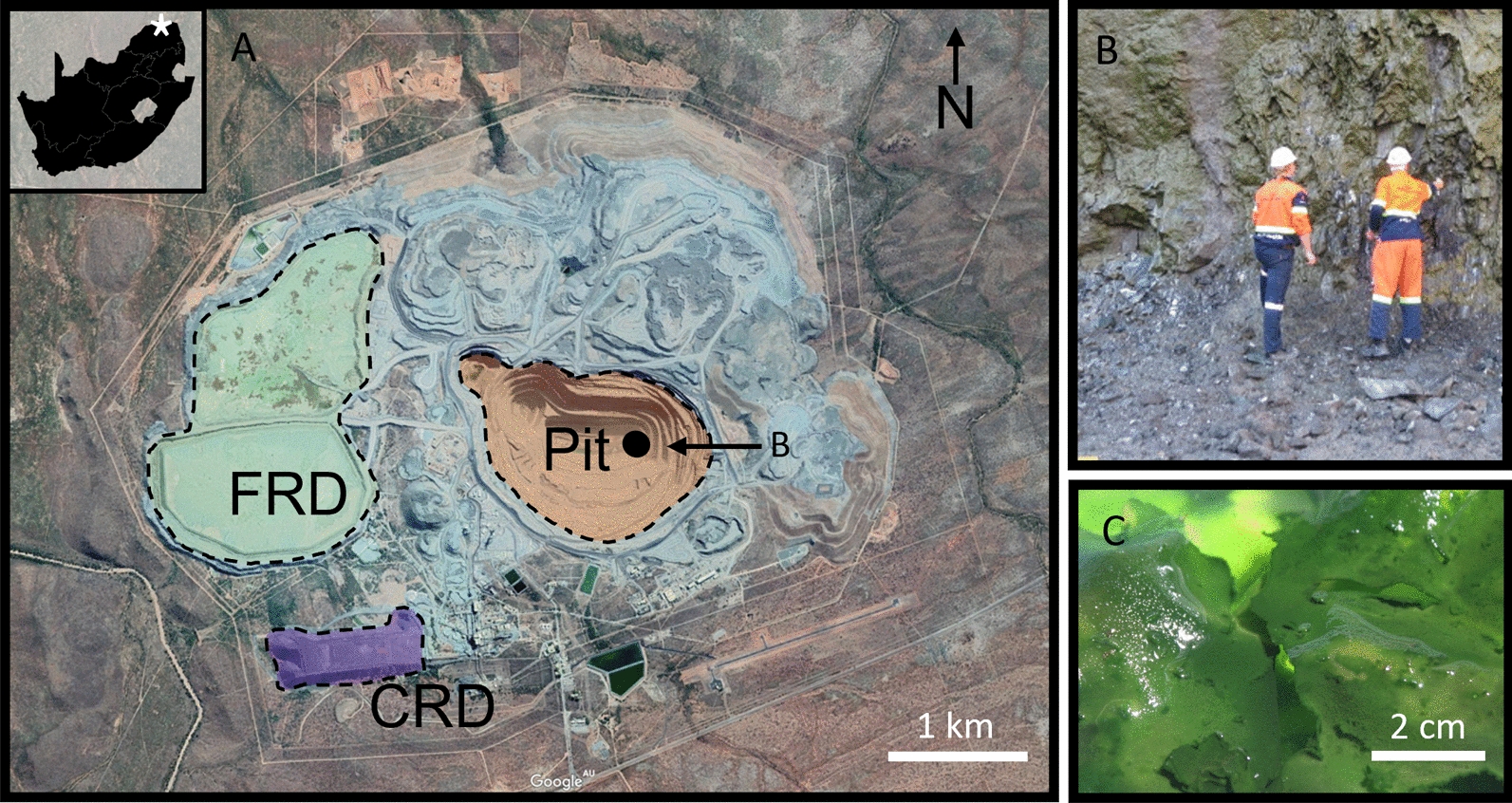
**A** Google Earth image of the Venetia diamond mine, Limpopo, South Africa, its location indicated by the star on the inset image (22°25′59'' S, 29°18′50''E) of South Africa. Coloured areas showing the layout of the primary investigation areas; the pit, CRD and FRD; **B** bacterial sampling on bench 29 and, **C** the microbial culture grown in BG-11 for 8 weeks

Industrial CO_2_ sequestration via mineral carbonation relies simply on the acceleration of these natural geological processes by increasing the activity of relevant microbial populations, the weathering of silicate minerals, and the transfer of suitable cations (Ca^2+^, Mg^2+^) from silicates to carbonate [15]. The engineering approach to mineral carbonation often involves crushing/pulverising, a leaching step (chemical additives), and CO_2_ capture and transport, all of which are costly, but may be accelerated inexpensively, via bacterially-driven processes acting on a suitable substrate [15, 26]. Mineral carbonation can be approached as in situ, and ex situ processes. *In-situ* systems involve the transport and injection of CO_2_, either as a supercritical fluid or aqueous solution (carbonic acid), into a suitable reactive host substrate, such as basalt or peridotite (Fig. 1a) [20]. Ex situ carbonation is the process of precipitating carbonate in a mined or altered feedstock. This may entail physical and chemical pre-treatments, such as crushing and acid-leaching [20, 30]. Carbonation via this process is controlled by the same factors as in situ carbonation, though the feedstock is more accessible and can be augmented in ways that aim to increase the potential for carbon storage.

Mineral carbonation is dependent on the available cations, a source of inorganic carbon and pH of the environment in which it is taking place. Therefore, the mineral substrate in which any proposed carbonation acceleration is to take place must be susceptible to weathering. Kimberlites are one such substrate, with well-known low-temperature alteration reactions—transforming unaltered kimberlite (blue ground) into a weathered/friable kimberlite (yellow ground), and the resulting carbon sequestration via the formation of secondary carbonate minerals [2, 7, 14, 25, 29]. This natural phenomenon, occurring in kimberlites exposed to Earth’s surface weathering conditions, could be accelerated as a by-product of extraction and crushing [19, 28].

This study is part of Project Carbon Vault™, an initiative set-up by the De Beers Group aimed at offsetting CO_2_ emissions associated with diamond mining. The ultramafic residue produced by extracting diamonds from kimberlite has the potential to sequester large amounts of CO_2_ through mineral carbonation [7, 12]. The aim of this study is to examine the impact of microbial activity on the degree of mineral carbonation in kimberlite, where changes in the biogeochemical weathering conditions increases the amount of carbonate precipitated over time.

## Materials and methods

### Field site, sample collection and bacterial culture work

The Venetia diamond mine is in Limpopo, South Africa (22° 25′ 59'' S, 29° 18′ 50'' E). Mining has been underway since 1992, during which 12 kimberlite occurrences have been discovered: 11 kimberlite ‘pipes’, and 1 intrusive dyke [1]. Venetia is South Africa’s largest producer of diamonds, extracting approximately 4.2 million carats in 2018. Processed samples from the mine are dominated by phyllosilicates, primarily serpentine minerals and smectites (19.0 wt% and 36.6 wt%, respectively), with diopside present at an average abundance of 20.5 wt% [12]. The footprint of the Venetia mine site is dominated by three reservoirs: the mine pit, the Fine Residue Deposit (FRD) and the Coarse Residue Deposit (CRD) (Fig. 1A). FRD and CRD refer to the two size fractions produced during kimberlite processing: FRD ≤ 800 μm grain size, with CRD ranging from 800 μm–8 mm in size. A 5 kg sample of FRD and a 5 kg sample of CRD were collected for laboratory experimentation at the University of Queensland.

A photosynthetic biofilm sample, collected from the vertical face along Bench 29 in the eastern portion of the pit (Fig. 1AB), was cultured for 4 weeks at 21 °C in 2 L BG11 medium [17] in a 15 cm X 20 cm X 5 cm deep plastic container receiving natural light and possessing 10 g/L FRD (Fig. 1C as a mineral nutrient and further inoculum; referred to as the lab. consortia.

### Experimental weathering columns

A column weathering experiment was developed to model the sequential deposition of CRD post processing: comprising a surface incubation phase, representing near surface depositional conditions, i.e., exposure to light, and a subsequent burial phase. Five experimental columns were set up in sterile 60 mlpolypropylene syringes fitted with ca. 1 cm^3^ glass wool at the bottom of the syringe to prevent loss of CRD or weathered products. CRD (38 g) and 2 ml of biofilm from the primary enrichment culture were added to each of the experimental columns as described below depending on whether the treatment would be exposed to surficial or burial-like conditions. Each column was then covered with a loose-fitting plastic cap to prevent dust contamination and was incubated at room temperature.

Each experimental system was fed weekly with 2 ml of BG-11 medium, pH 7, creating wetting and drying conditions. This volume kept the system wet while limiting the loss of liquid from the bottom of the columns, thereby minimising the amount of soluble materials flushed out of the system. An uninoculated control was prepared in the same manner as above and was fed 2 ml of sterile water weekly. This uninoculated control was kept under dark, burial conditions (see “Burial conditions” section).

#### Surficial conditions

The surficial experimental systems (n = 2) were run using two inoculation methods. A ‘sprayed’ inoculation, i.e., a ‘top-down’ inoculation-culturing method, of introducing biofilm to the surface of the CRD after deposition. This was compared to a ‘mixed’ inoculation where the biofilm and CRD were combined in a 50 ml Falcon tube and dispersed using a vortex mixer for 20 s prior loading it into the 60 ml polypropylene syringe. This mixing step produced a ‘damp’ microbe-mineral slurry consistent with mixing during conveyor transport to the CRD pile, i.e., prior to deposition. The surficial experiments were exposed to natural light conditions (a natural 24 h day/night cycle) for the duration of the 12-month experiment.

#### Burial conditions

The burial experimental systems (n = 2) were both run using the ‘mixed’ inoculation method as described above (See “Surficial conditions”). Two systems were developed to assess the effect of vadose and saturated conditions on mineral carbonation. The vadose experiment remained open to the atmosphere at the top and bottom of the syringe. The saturated experiment was fitted with a stopper to prevent the loss of liquid from the system and was kept submerged. Both burial condition experiments were exposed to natural light for 4 weeks to initially support the growth of the photosynthetic biofilm inoculum, before being deprived of light, consistent with burial, for a further 12 months. Light deprivation was achieved by wrapping each system in aluminium foil and storing in a cupboard. After incubation, a vertical one-half section of the column was removed for carbon and DNA analyses, X-ray diffraction (XRD), scanning electron microscopy (SEM), and embedding/synchrotron X-ray florescence microscopy (XFM); described individually below.

### Carbon analyses

A composite sample (10 g) of each experiment was removed from across the entire height of the column (preserving one half of each column for embedding, below), and oven dried @ 45 °C for 1 week, then crushed using an agate mill to approximately 100 µm grain size. These samples were split and sent for Total/Inorganic/Organic Carbon analysis (TC/TIC/TOC). The carbon samples were analysed at the School of Agriculture and Food Sciences, UQ, by combustion using a LECO 928 analyser.

### DNA analyses

One gram samples from each system were taken for DNA analysis. Before sampling, any floating biofilm that had detached from the kimberlite surface by entrapped oxygen bubbles was removed as it was unlikely these photosynthetic microbial mats had a high degree of kimberlite rock interaction. Note, this does not equally infer that the kimberlite did not promote biofilm growth, as limiting nutrients, e.g., phosphorous, or trace nutrients, e.g., iron, likely promoted biofilm formation. The samples were taken using a sterile spatula and consisted of sub-subsamples from the upper (1–2 cm), middle (~ 5 cm), and lower (~ 10 cm) sections of the syringe columns–with all work being undertaken in a sterile laminar flow cabinet. Samples were submerged in LifeGuard^®^ Soil Preservation Solution (Qiagen) before DNA extraction, amplification of 16S rDNA genes and sequencing at the Australian Centre for Ecogenomics. DNA was extracted from each of the samples using 0.1 cm diameter glass beads for preliminary bead beating (BioSpec Products #11079101) on a Powerlyser 24 homogenizer. The samples were then added to a bead tube filled with 850 µl of CD1 (Qiagen cat #47016) to be mixed by vortex. Tubes were heated to 65° C for 10 min and then beaten for 5 min at 2000 RPM, before a final centrifuge for one minute at 15 000 g. The resulting lysate was then transferred to a new collection tube for PCR amplification with a final elution volume of 50 µl. Amplification of the V6 to V8 regions of the 16 s rRNA gene using the universal primers 926f and 1392r adapted to contain Illumina-specific adapter sequences (adapter sequences in capitals): 926F: 5′-TCGTCGGCAGCGTCAGATGTGTATAAGAGACAGaaactyaaakgaattgacgg-3′ and 1392wR:5′GTCTCGTGGGCTCGGGTCTCGTGGGCTCGGAGATGTGTATAAGAGACAGacgggcggtgtgtrc-3′. Libraries were prepared as described by Illumina (#15044223 Rev B), except that Q5 Hot Start High-Fidelity polymerase and PCR mastermix were used (New England Biolabs, Ipswich, MA, USA). PCR amplicons were purified using Agencourt AMPure XP beads (Beckman Coulter, Brea, CA, USA). Purified DNA was indexed with unique 8-bp barcodes using the Illumina Nextera XT 384 Index Kit A-D (Illumina FC-131–1002) in standard PCR conditions with NEBNext® Ultra™ II Q5® Mastermix. Indexed amplicons were pooled together in equimolar concentrations and sequenced on a MiSeq Sequencing System (Illumina) using paired end sequencing with V3 300 bp chemistry in accordance with the manufacturer’s protocol at the Australian Centre for Ecogenomics—the University of Queensland.

Sequences were processed using the method by Gagen et al. [6] except with updated MOTHUR Version 1.45.3 [21] and the updated SILVA v138 database [16]. Sequences were grouped into operational taxonomic units (OTUs) to 97% similarity. Representative sequences of major OTUs were compared to publicly available sequences using the Nucleotide Basic Local Alignment Search Tool (BLASTn) at the NCBI and the non-redundant nucleotide collection, excluding uncultured selections.

### Secondary electron scanning electron microscopy (SEM)

Material was removed from the top one cm of each experiment to prevent damage to the remaining column (embedding below), and fixed @ 4 °C for 24 h in 2.5% glutaraldehyde for electron microscopy. All samples were then dehydrated using an ethanol dehydration series (20%, 40%, 60%, 80%, 3 X 100%), and dried using a hexamethyldisilizane series with 100% ethanol (1:3, 2:2, 3:1, 3:0). Prior to microscopy, samples were placed in a vacuum oven overnight @ 40 °C, plasma cleaned (Evactron Plasma Cleaner) and carbon coated (Quorum Q150T carbon coater). Field Emission Scanning Electron Microscopy (FE-SEM) was performed using a JEOLJSM – 7100F at an accelerating voltage of 20 keV for back scattered and between 2 – 4 keV for secondary electron imaging, with a working distance of 10 mm.

### Embedding and thick-section preparation.

The remaining material left in-situ within the syringes was oven dried at 40 °C for 24 h before being dehydrated using 100% ethanol. Samples were embedded in the syringe, sequentially, with two resins. LR White resin was used initially as it was unlikely the samples were completely void of water; this resin was allowed to flow through the samples three times prior to setting at 60 °C for 48 h. Prior to setting, the samples were capped and sealed to limit the contact of oxygen with the resin while polymerization occurred. Once hardened, Epo-Tek 301 resin was used to further ensure the rigidity and strength required for sample polishing. Each of the samples were then cut and formed into sections approximately 150 µm thick to ensure the stability of the biofilm.

### Synchrotron

Elemental distributions of the 150 µm thick samples were mapped using the XFM Beamline at the Australian Synchrotron, Clayton, Australia [13]. A monochromatic 12.9 keV X-ray beam was focused to 2 µm using a Kirkpatrick-Baez mirror pair. Overview scans were run with 20 µm pixels, which were decreased to 2 µm for high resolution maps captured by a 384 element Maia detector [22]. Image data was analysed using GeoPIXE [18] to produce elemental maps of the experimental systems.

## Results

### Total carbon* (TC)/*total inorganic carbon* (TIC)/*total organic carbon* (TOC)*

**Fig. 2:**
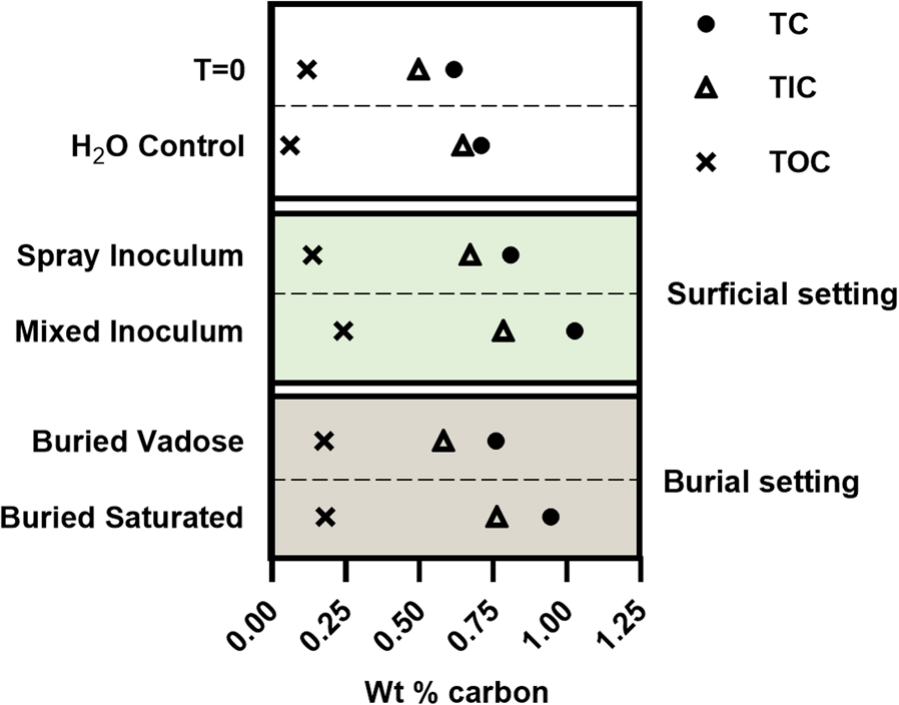
Weight percentage of carbon in each experimental setting after one year incubation versus the T = 0 values, which represents the initial carbon contents of the kimberlite CRD

When incubated under surface conditions (i.e., exposed to light), both inoculation methods (spray and mixed) increased the amounts of TC, TIC and TOC, relative to the uninoculated H_2_O control demonstrating that inoculation enhanced carbon sequestration. The mixed inoculation method resulted in the highest wt. % increase for all carbon species (Fig. 2; 0.41 wt% TC increase), versus the spray inoculation (0.19% wt increase) which was approximately double the uninoculated H_2_O surface condition control (0.09 wt% increase). Experiments incubated under a burial setting yielded interesting results, with vadose conditions producing minimal carbonate and the saturated conditions producing close to the same amount of carbonate as the mixed inoculum incubated under surface conditions (Fig. 2; burial TC increase of 0.33%). Interestingly, the increased wt% TIC in the uninoculated H_2_O control corresponded to a decrease in TOC suggesting that growth of endogenous bacteria from the kimberlite on natural organic carbon may have supported mineral carbonation.

### Bacterial population dynamics

**Fig. 3:**
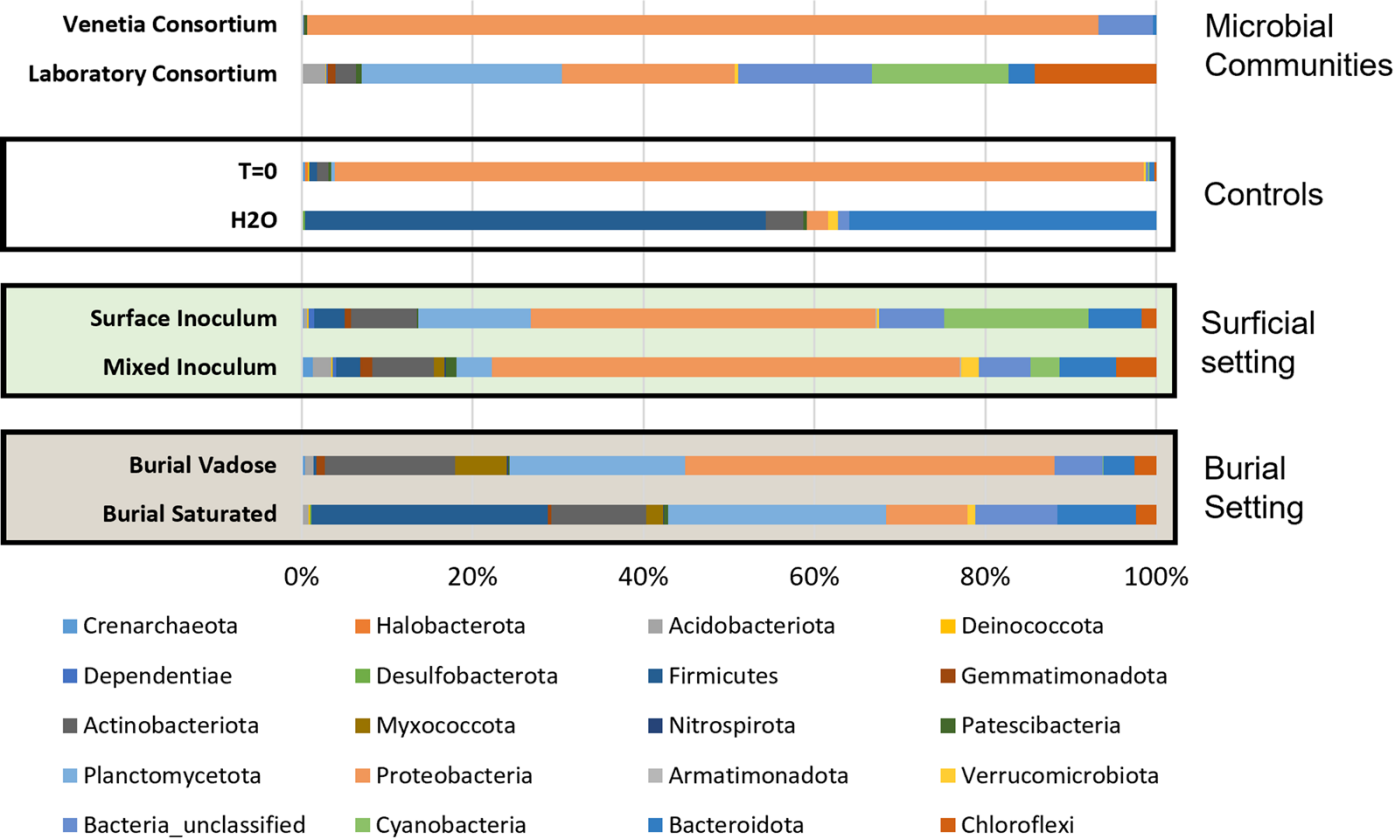
Relative abundance and phylum level classification of bacterial and archaeal Fig. 3: Relative abundance of bacteria within each system. The Venetia consortium represents the original biofilm sampled from the Venetia mine pit and the lab. consortia shows the same community after being grown in a BG-11 medium. T = 0 shows the bacterial community present in the CRD material prior to inoculation. The H2O control shows the microbiome within the CRD after one year of weekly feedings using DI water. Primary difference between the surficial and burial settings is the presence and absence of cyanobacteria. A 0.01% population cut-off was used to reduced noise

The microbial consortia inhabiting the different systems reflect the incubation conditions consistent with the principal population differences being driven by light. Gross microbial changes (Fig. 3) at the phylum level reveal the presence, or lack thereof, of any significant populations of cyanobacteria within the different systems. From this data, and as expected, the presence of light promotes the growth of cyanobacterial populations that are otherwise not seen when a sufficient light source is not provided. Surficial inoculation was shown to allow for greater growth of cyanobacteria (16.8% of total sequences) as opposed to 3.3% relative abundance in the mixed inoculation experiment. This was an expected outcome as the surficial conditions allowed for growth unimpeded by overlaying kimberlite material as was the case in the mixed experiment.

**Fig. 4:**
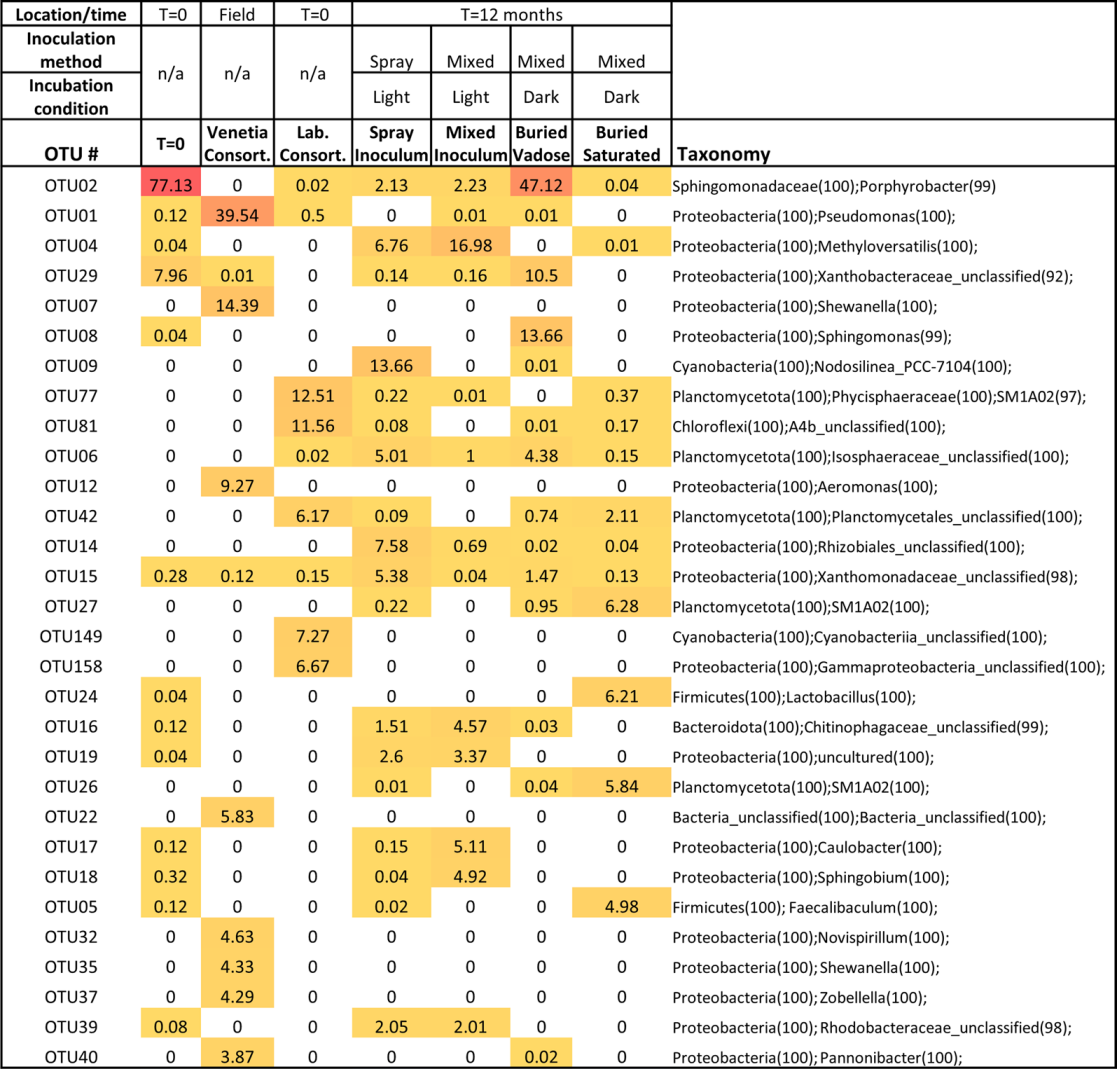
Heatmap denoting abundance of major bacterial OTUs in each sample. Darker colours (red) represent higher abundance while light colours (white) represent lower abundance. Taxonomy, with confidence shown in brackets, has been shown at phylum level and then only at the highest resolution thereafter

A higher resolution observation of the changes in microbial community and Genera levels reveals distinct changes between the Venetia consortia that was sampled from the active pit at the Venetia diamond mine, and the lab. consortia that grew from the collected sample (Fig. 4). The original microbes present in the Venetia consortia were not present in any meaningful abundance after growth lab setting, and throughout the subsequent experiments. The lab. consortia aligns significantly with the microbial community isolated from the T = 0 CRD material (Fig. 4), the same material that was added to the lab growth bioreactor to enhance growth of the biofilm to be used in the experiments. Additionally, the Cyanobacteria present in the lab and experimental biofilms are different to that found in the original Venetia consortia.

### Calcium and carbonate tracking

**Fig. 5:**
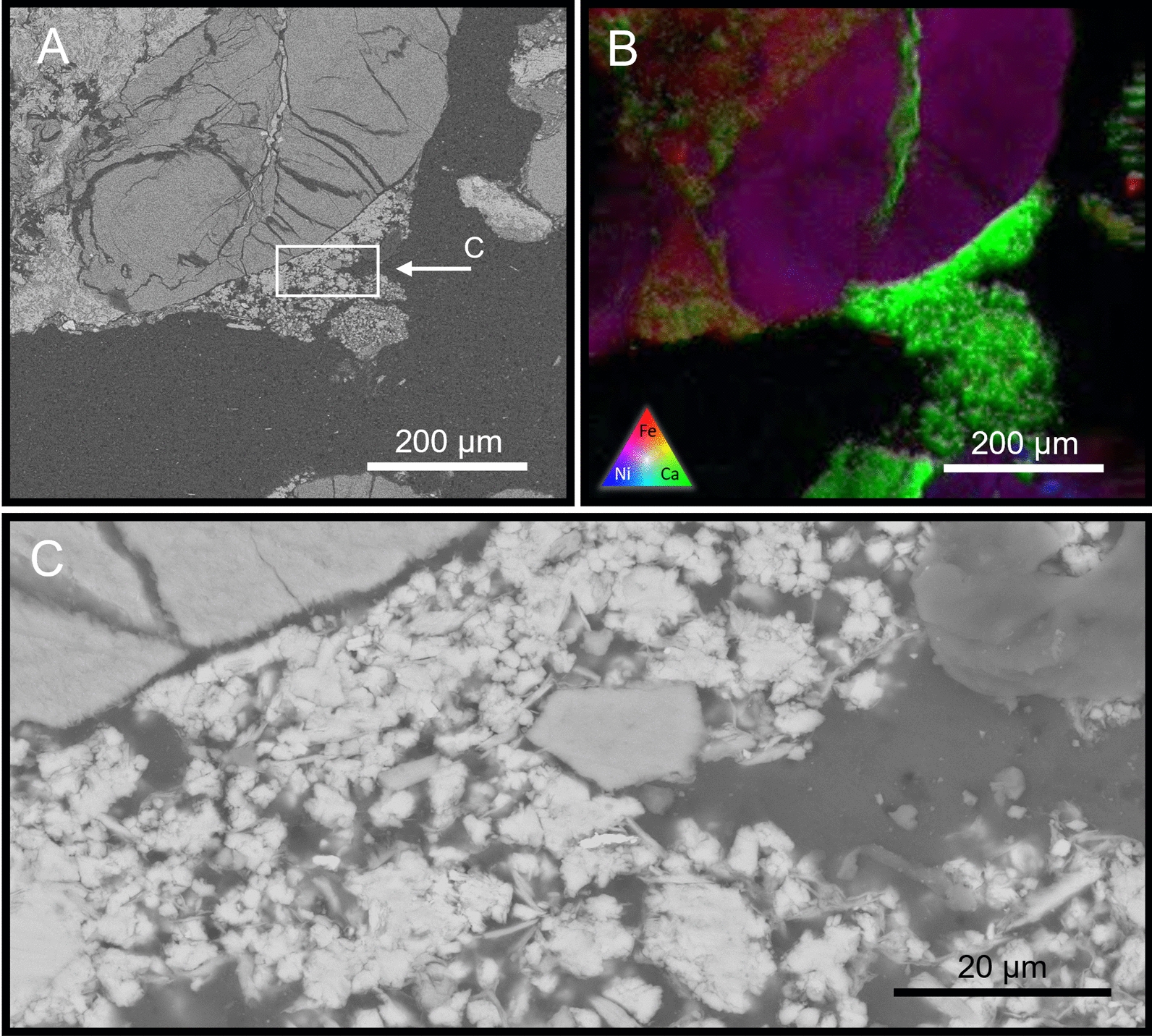
Back-scatter SEM (**A**) and XFM (**B**) of the mixed inoculation surficial experiment showing highly weathered primary mineralogy of the CRD. Calcium-rich secondary minerals (**B**—green) are seen precipitating between discreet minerals, forming a linking cement. High resolution BSE-SEM (**C**; EDS data not shown) suggest calcium carbonate minerals with a large number of individual nucleation sites

Low resolution (20 μm—pixel) XFM elemental maps were produced to screen the cm-scale samples to identify calcium rich areas that were to be investigated using high resolution mapping for primary versus secondary mineralogy. Representative areas paired with backscattered electron SEM were used to demonstrate the occurrence of calcium rich secondary minerals located throughout the columns. Both surficial experiments possessed secondary calcium-bearing minerals, often at grain boundaries, that were shown to be carbonates based on EDS ((Ca–C–O); Fig. 5). This carbonate was scattered throughout the samples, often in clumps ranging between 200 μm and 500 μm in diameter and bridging between primary kimberlite grains, producing the initial stages of intergranular cementation.

**Fig. 6:**
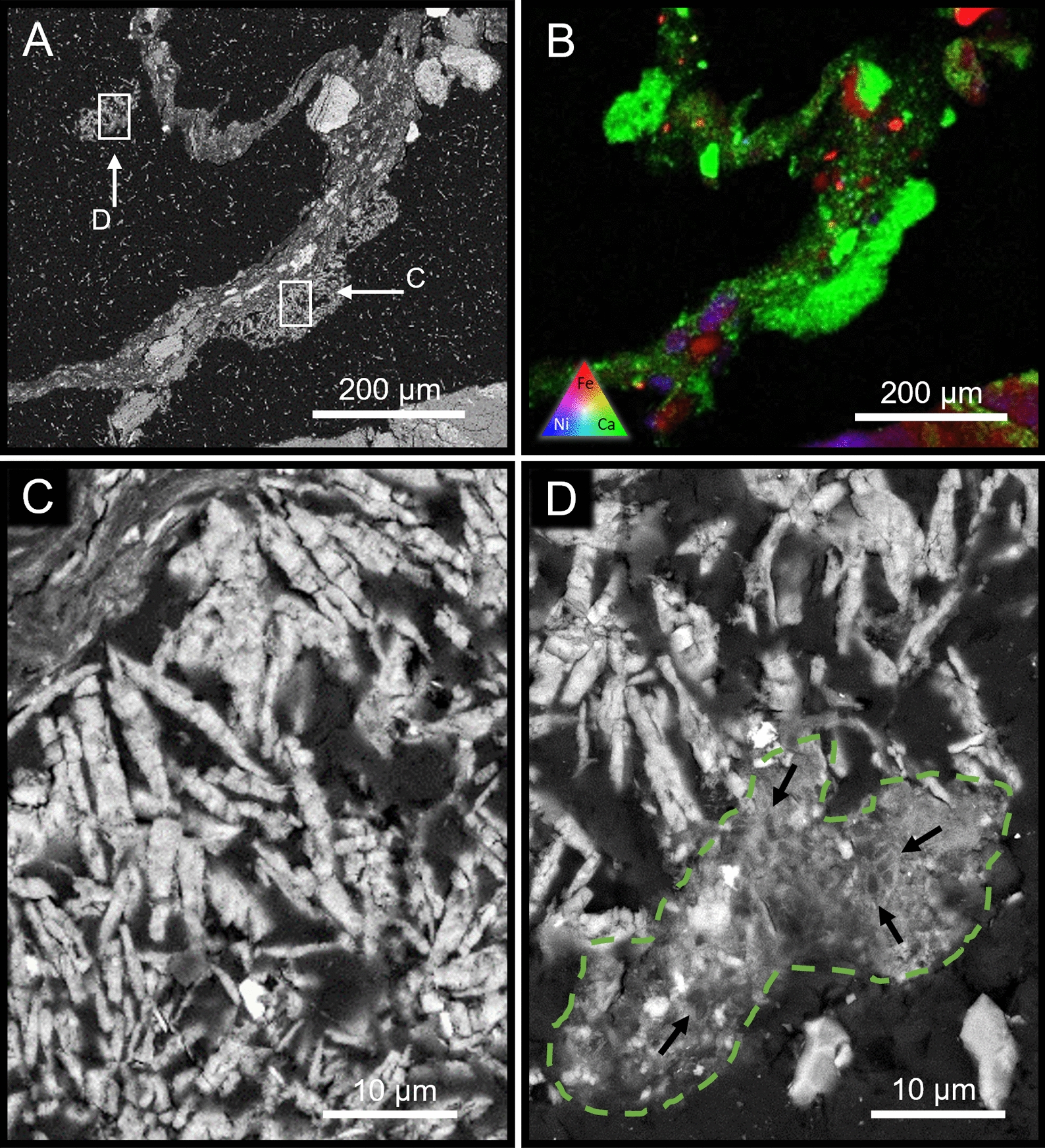
BSE-SEM micrograph (**A**) and XFM map (**B**) of the vadose experiment incubated under burial (dark) conditions highlighting the occurrence of an extensive amount of fine-grained secondary minerals and weathered CRD grains held within a sample of biofilm. At high magnification (BSE-SEM), the bladed crystals of the Ca-S–O evaporite (**C** and **D**) suggest gypsum (EDS data not shown) and Ca-O-(C) is consistent with calcium carbonate. A high magnification BSE-SEM image highlighted in tile (**D**), amorphous carbonate precipitates are present in the system, though predominately seen in association with sulphate minerals (**D**). Small arrows focus on the locations of permineralised bacteria

The burial experiments both produced carbonates, though likely via different, i.e., heterotrophic mechanisms given that they were incubated under dark conditions. Using a combination of XFM mapping (Fig. 6) and confirmed via Back-scatter SEM–EDS, large areas of secondary calcium sulphate minerals were observed throughout the vadose burial experiment. This mineralogy was not observed in any other experimental system. This buried vadose system produced the least amount of secondary carbonate, in part due to the preferential, abiotic precipitation of calcium sulphate minerals. However small ‘pockets’ of anhedral, biogenic carbonate were observed throughout the system. These biogenic carbonate clusters, possessing fossilized bacteria, were only found in association with the abiotic gypsum precipitates (Fig. 6D) suggesting that transformation from calcium-sulphate to calcium carbonate is possible with biology.

**Fig. 7:**
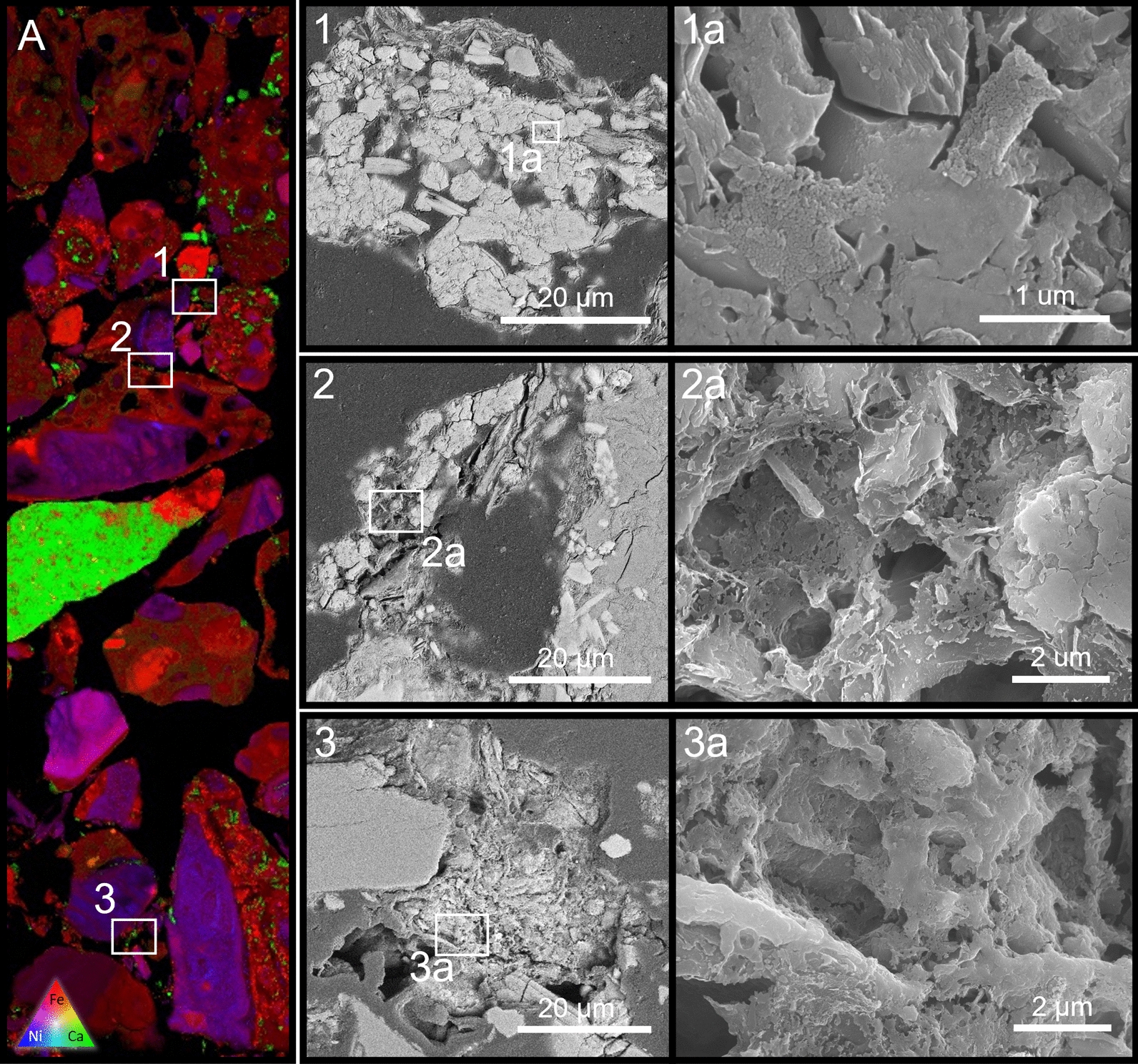
XFM map (**A**) showing an embedded section of the saturated burial phase experiment. Panels 1, 2, & 3 on the map highlight the presence of calcium bearing secondary carbonate minerals growing on weathered grains boundaries of the primary minerals cementing the grains together. Multiple nucleation sites are visible within the larger (ca. 50 μm) carbonate minerals (see panels 1, 2 and 3). Tile 1a shows the presence of nano scale (10 nm) amorphous carbonate nodules covering the surface of more well-developed carbonate minerals. Lightly etched sections of carbonate (panels 2**a** and 3**a**) reveal underlying rod-shaped bacteria embedded within the cement. Poor level of microbial preservation due to the multiple drying and embedding treatments involved in the creation of the thin sections

The burial, saturation experiment produced carbonate minerals resembling that produced in the surface experiments, though they occurred as smaller, mineralised bacterial microcolonies. Still occurring as intergranular ‘cements’, they were only seen as round clumps up to 100 μm in diameter (Fig. 7, panels 1, 2, and 3). There is also evidence of 100 nm-scale ‘amorphous’ carbonate mineral precipitates in association with the larger carbonate grains (Fig. 7, panel 1a).

**Fig. 8:**
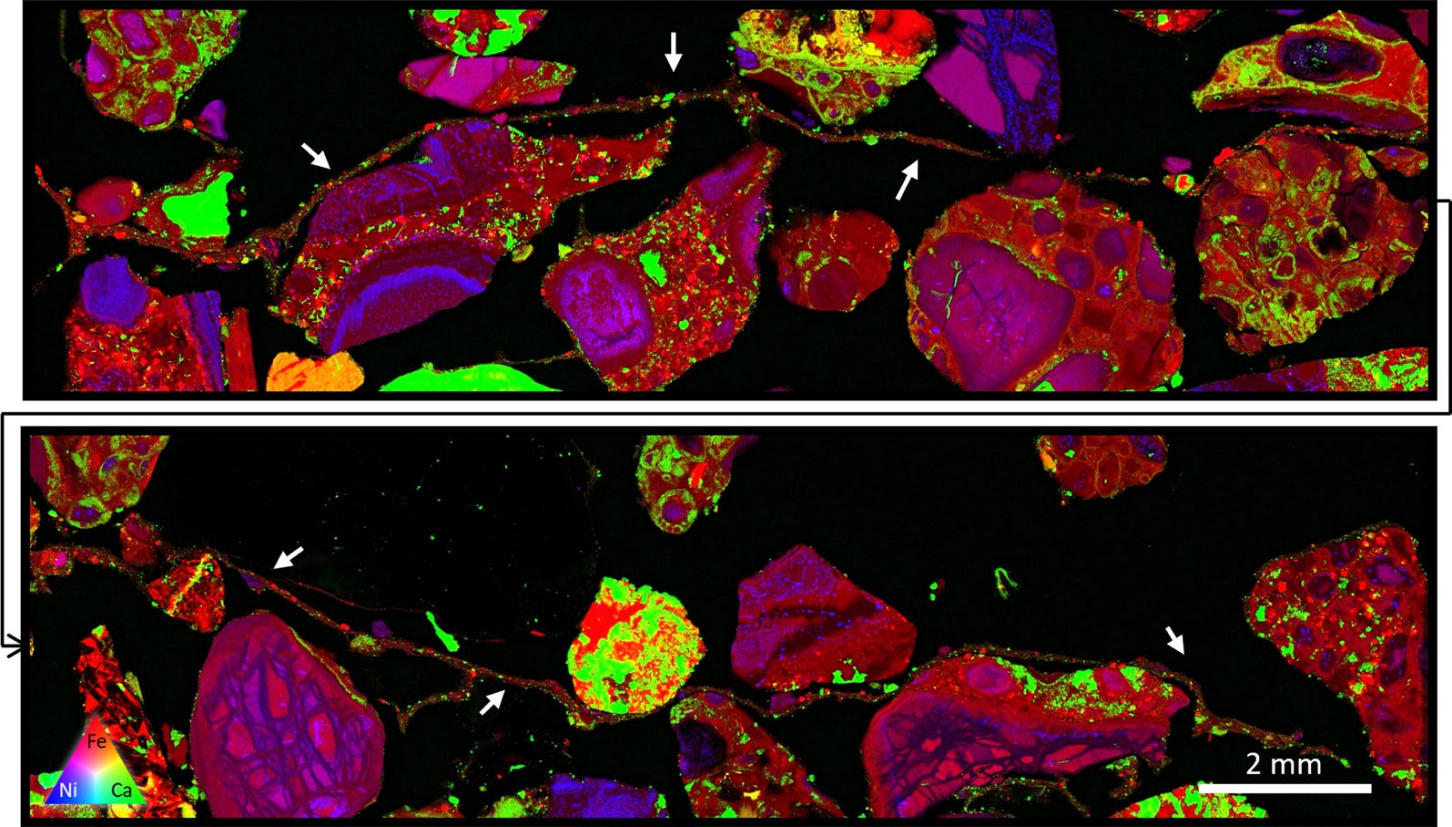
A split XFM image showing a continuous biofilm traversing numerous CRD grains. The extent of the biofilm (predominantly represented in red, likely where Fe particles have been trapped in the biofilm) extends around 30 mm in length (location emphasized by arrows). As carbonate precipitation within these systems is driven by the presence of the microbes, the intergranular growth of the biofilm works to directly accelerate the development of intergranular carbonate

The extent of the microbial growth across these systems is shown in Fig. 8, where a biofilm is shown traversing across multiple discrete grains of the CRD material.

## Discussion

### Carbon sink

In a recent study of carbon sequestration potential of the Venetia mine in South Africa, 0.21 MT of CO_2_e represented the offset required to counter annual emissions [12]. Using the optimum increase in TC in the mixed inoculum photosynthetic column (0.41 wt%) and converting this value to CO_2_e for the estimated 4.74 MT of kimberlite processed each year, demonstrated that 34% of mine emissions could be offset. The inclusion of TOC in this calculation is based on the observation from the water control that a decreased TOC value corresponded to an increased TIC measure, a conversion likely catalysed heterotrophic bacteria (Fig. 2). A reality associated with CRD waste disposal, is that layering new materials on the waste pile will transform material from an active photic zone to a burial condition. However, using the saturated burial value of 0.33 wt% TC, 27% of annual mine emissions are still offset (Table 1).

**Table 1:**
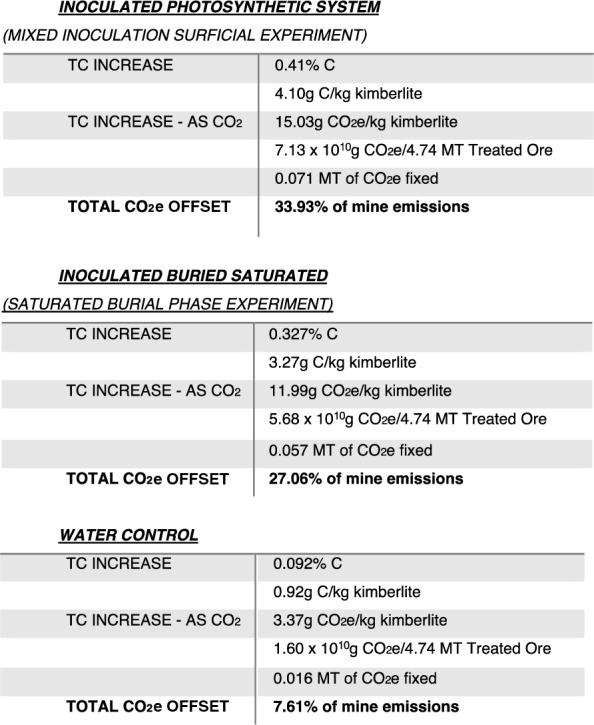
CO_2_e offset equivalents

The sequestration capacity calculation from Mervine et al. [12] is based on an average mineralogy of the Venetia kimberlites, whereby the Ca, Mg, Fe, and Mn, primarily bound in silicate, oxide and hydroxide minerals are assumed to be liberated and available for precipitation with atmospheric CO_2_. However, the measurement of only Ca and Mg in the secondary carbonates observed in this study (Figs. 5, 6, 7 and 8) suggests that this original estimate-capacity should not include Fe or Mn-carbonate products in the mineral carbonation capacity until weathering and carbonation of these elements/compounds are demonstrated.

### Vadose conditions

While the buried, vadose experiment was not particularly successful from a mineral carbonation perspective, it demonstrated the critical importance of biology in mineral carbonation, i.e., secondary carbonate was only formed in association with bacteria. The small pockets of secondary carbonate observed within the vadose burial system all possessed fossilised bacteria (Fig. 6D). The formation of secondary calcium sulphate minerals suggests that these systems possessed little water. While the likely occurrence of gypsum indicates that primary mineral dissolution is occurring, releasing calcium and sulphur into the environment, the system appears to be carbon starved preventing calcium from forming carbonates even though the samples were ‘directly’ exposed to atmospheric CO_2_ under vadose conditions. The source of this sulphur likely stems from the weathering of millerite (NiS) which was observed sparingly throughout the kimberlite mineralogy, along with primary small amounts of pre-existing gypsum, likely associated with smectites [30].

In contrast, the mixed photosynthetic experiment was also incubated under vadose conditions, but the precipitation of sulphates was not observed. This was likely due, in part, to the increased biomass and exopolymeric substances formed under surface conditions allowing for higher water retention, limiting evaporite formation, and the dominance of cyanobacteria, generating alkalinity, which would preferentially form carbonate minerals (Eq. 3).

### Microbial role in weathering and precipitation

Weathering of primary, blue ground mineralogy is a prerequisite for mineral carbonation and, given the enhanced biogeochemical weathering observed in this study, the activity of the microorganisms within the system must play a role in the breakdown of the kimberlite and geochemical cycling. While kimberlite can be extremely heterogenous across a single pipe [5, 12], the weathered consistency of yellow ground, relative to the background soil matrix, in near-surface kimberlites suggest that weathering of these materials just like soil formation must provide some nutritional benefit to promote microbially enhanced weathering, e.g., the TOC at T = 0. This microbial factor (bacterial metabolism) facilitated by their need for nutrients accelerated weathering of primary minerals releasing inorganic and presumably organic materials into solution, forming secondary mineral products.

Microbial carbonate precipitation occurred in both the photic columns and those deprived of light, indicating that reactions other than those driven by photosynthesis may also contribute to weathering and mineral carbonation. For example, the microenvironments dominated by Ca-sulphate mineralogy suggests the importance of sulphate reducing bacteria (though none observed in the most abundant OTU’s presented in Fig. 4) in producing carbonate mineralogy via the reduction of sulphate to hydrogen sulphide:

7 $${{{C}_{2}{H}_{4}O}_{2}}_{(l)}+ {{SO}_{4}^{2-} }_{(aq)}\to {{H}_{2}S}_{(g)}+{{2HCO}_{3}^{-}}_{(aq)}$$

Microbial sulphate reduction in anaerobic environments produces chemistry that can trend toward carbonate precipitation. This has been observed in modern marine stromatolites and cyanobacterial mats, where carbonate laminae precipitate in zones of active sulphate reduction [9, 11, 27]**.** The level of saturation within the environment evidently plays a large role in in the carbonation efficiency of CRD once it becomes buried with subsequent material—with the higher saturation leading to an increase in secondary carbonate precipitation. The saturated environment (Fig. 7) did not result in the occurrence of gypsum evaporites at 1 year incubation, possibly supporting carbon sequestration/mineral carbonation by dissimilatory sulphate reduction preventing gypsum formation. The cation exchange capacity of the smectite within these samples will also be an ongoing reaction likely occurring at a relatively similar rate across the different systems. This reaction acts as a further source of cations for carbonation [30].

The growth of the Venetia consortia in the lab significantly shifted the population away from the originally sampled microbes, and towards that seen in the T = 0 CRD material that was used in the bioreactor to apply environmental pressure and aid in biomass growth. The resulting lab. consortia that was used in the column experiments contained microbes present in both the Venetia consortia and the CRD material, along with microbes not prominent in either.

The bacterial enrichment culture and the corresponding biogeochemical weathering has initiated an early stage in yellow ground formation, beginning the transformation of the unweathered CRD blue ground kimberlite into a soil.

### Biotechnology: the Venetia CRD deposits as a microbially driven carbon sink.

The Venetia mine produces 4.74 Mt of residue (treated ore) per year, a combination of both 40% CRD and 60% Fine Residue Deposit (FRD) [12]. The biogeochemical conditions occurring in CRD, i.e., crushed kimberlite, will vary macroscopically across layers and microscopically based on water saturation and oxygen/CO_2_ ‘penetration’—affecting biogeochemical weathering. The surficial and burial phases of the CRD depositional cycle differ in environmental conditions, though remarkably, each process resulted a 34% and a 27% annual offset, respectively (based on total processed kimberlite).

The mixed inoculum photosynthetic treatment of the CRD produced the highest autotrophic CO_2_ fixation and carbonate precipitation, totalling 34% of annual CO_2_e, which corresponds to the precipitation of 178,145 tons of calcium carbonate per year, if the total processed kimberlite could achieve the same level of mineral carbonation. Similarly, if the measured burial reactivity, corresponding to 142,081 tons calcium carbonate in one year, can continue beyond the 1-year incubation employed in this study, then much higher mineral carbonation levels can ultimately be reached. Mineral carbonation dependent on cations derived from its host material can be susceptible to passivation, which would work to lower a systems carbonation potential. This affect is eased within most kimberlite systems due to the large presence of Ca-Mg bearing, highly reactive smectites, with high cation exchange capacities [30]. Mineral carbonation occurring naturally in other diamond mine residue facilities, such as those present at the Cullinan diamond mine, does not show evidence of carbonate passivation. These 50-year-old residue deposits show continued carbonation via ongoing mineral breakdown, even with the system wide development of secondary carbonate mineralogy covering nearly all the discrete grains [7].

The Venetia CRD is a vast deposit of processed material, which if treated under conditions for biologically accelerated weathering, i.e., mineral dissolution and mineral carbonation, can act as a large-scale carbon sink requiring very little intervention, i.e., simply requiring the growth of photosynthetic biofilm in this ambient, sunny climate, followed by inoculation of CRD tailings. The inoculation of kimberlite via mixing is recommended, which would see the bacteria dispersed through the material, exposing the highest amount of materials to the microbes. This higher degree of microbe-mineral interaction increases the potential for biogeochemical weathering, promotion of CO_2_ access, and therefore, enhanced carbonation (Table 1). A suggested site for inoculation of crushed kimberlite material would be on the conveyor belt whilst being transported for deposition.

While photosynthetically-driven near surface biogeochemical carbonation reactions were optimal in the laboratory-scale experiment (Fig. 2), it is impractical to expose this degree of mine waste at a decimetre depth. Given the massive amount of waste materials produced by diamond mines [12], burial in CRD piles will represent the more common occurrence of these materials. Under burial conditions, the saturated environment proved to be far more conducive to the precipitation of secondary carbonate material than the vadose environment. However, the reduced biogenic carbonation observed in the dark, vadose condition may simply require more time to achieve optimal (complete) mineral carbonation.

## Conclusion

The potential for carbon mineralisation within mined kimberlitic material was enhanced by microbial inoculation, with the addition of microbes increasing weathering and the deposition of secondary, calcium and magnesium carbonates. Based on the formation of Ca-Mg carbonates, this accelerated carbonation was likely due to the breakdown of Ca-Mg-bearing silicates or cation exchange of clays [30], releasing the needed cations for carbonation.

The microbiome that developed in the burial treatments of this experiment is essential to this process because the amount of photosynthetic activity will be limited to the surface of the CRD deposit, i.e., light exposure; however, large amounts of carbonation can still be achieved in the dark (Fig. 2). How these rates vary over time and with continued burial is unknown, though ongoing mineral carbonation has been observed in much older residue deposits at other locals [7]. Long-term management of the Venetia residue could allow for continued precipitation of carbonate within the kimberlite matrix, extending indefinitely for the life of mine, and after production ceases.

This simple method for biologically-accelerated mineral carbonation has further potential, and with ongoing optimisation and refinement in carbonation mechanisms, a greater reduction in the Venetia mine carbon footprint can be achieved. This experiment shows the importance of a long-term approach to the handling of crushed kimberlite material, and the establishment of environments that can facilitate beneficial biogeochemical processes in a given material over the long-term, whether that be carbon mineralisation, tailings stabilisation, controlling mine drainage or soil regeneration—though further studies are essential.

## Data Availability

Generally, not applicable/contact corresponding author.
